# Novel Machine Learning Approach for DDoS Cloud Detection: Bayesian-Based CNN and Data Fusion Enhancements

**DOI:** 10.3390/s24051418

**Published:** 2024-02-22

**Authors:** Ibtihal AlSaleh, Aida Al-Samawi, Liyth Nissirat

**Affiliations:** College of Computer Sciences and Information Technology, Department of Computer Networks, King Faisal University, Al-Ahsa 31982, Saudi Arabia; 221445332@student.kfu.edu.sa (I.A.); lnissirat@kfu.edu.sa (L.N.)

**Keywords:** cloud computing, cybersecurity risks, DDoS, machine learning, BaysCNN model, cloud detection, dimension reduction

## Abstract

Cloud computing has revolutionized the information technology landscape, offering businesses the flexibility to adapt to diverse business models without the need for costly on-site servers and network infrastructure. A recent survey reveals that 95% of enterprises have already embraced cloud technology, with 79% of their workloads migrating to cloud environments. However, the deployment of cloud technology introduces significant cybersecurity risks, including network security vulnerabilities, data access control challenges, and the ever-looming threat of cyber-attacks such as Distributed Denial of Service (DDoS) attacks, which pose substantial risks to both cloud and network security. While Intrusion Detection Systems (IDS) have traditionally been employed for DDoS attack detection, prior studies have been constrained by various limitations. In response to these challenges, we present an innovative machine learning approach for DDoS cloud detection, known as the Bayesian-based Convolutional Neural Network (BaysCNN) model. Leveraging the CICDDoS2019 dataset, which encompasses 88 features, we employ Principal Component Analysis (PCA) for dimensionality reduction. Our BaysCNN model comprises 19 layers of analysis, forming the basis for training and validation. Our experimental findings conclusively demonstrate that the BaysCNN model significantly enhances the accuracy of DDoS cloud detection, achieving an impressive average accuracy rate of 99.66% across 13 multi-class attacks. To further elevate the model’s performance, we introduce the Data Fusion BaysFusCNN approach, encompassing 27 layers. By leveraging Bayesian methods to estimate uncertainties and integrating features from multiple sources, this approach attains an even higher average accuracy of 99.79% across the same 13 multi-class attacks. Our proposed methodology not only offers valuable insights for the development of robust machine learning-based intrusion detection systems but also enhances the reliability and scalability of IDS in cloud computing environments. This empowers organizations to proactively mitigate security risks and fortify their defenses against malicious cyber-attacks.

## 1. Introduction

Cloud computing has ushered in a new era in information technology, providing businesses with cost-effective and flexible solutions for data storage, application hosting, and network services [1]. The paradigm shift towards cloud-based services has led to increased efficiency, scalability, and reduced infrastructure costs, making it an attractive option for organizations.

However, this widespread adoption of cloud technology has brought forth significant cybersecurity challenges. With vast amounts of data, high traffic volumes, the utilization of virtual machines (VMs), and concerns regarding data confidentiality, cloud environments have become prime targets for malicious actors [2].

One of the most severe threats facing cloud computing today is Distributed Denial of Service (DDoS) attacks. These attacks are designed to overwhelm a target’s resources, rendering it inaccessible to legitimate users and causing disruption to cloud services [3]. Detecting and mitigating DDoS attacks is paramount to ensuring the continued operation and security of cloud-based businesses.

Traditional intrusion detection systems have relied on statistical models for threat detection. However, these models often struggle to adapt to the dynamic and rapidly changing network environments inherent in cloud computing. They are also limited in their ability to address the unique challenges posed by DDoS attacks [4].

In response to these limitations, researchers have turned to advanced machine learning and deep learning techniques, particularly neural networks, to develop context-aware prediction models for DDoS attack detection and prediction [5]. Yet, existing methods in the literature face significant challenges when it comes to distinguishing application-layer DDoS attacks from legitimate traffic and relying on outdated datasets for training [6].

This study aims to bridge these gaps by introducing a novel machine learning approach for DDoS cloud detection: the Bayesian-based CNN (BaysCNN) model, coupled with data fusion enhancements. Leveraging the valuable CICDDoS2019 dataset, this research seeks to advance the field by addressing key challenges in DDoS detection in cloud computing environments.

To underscore the significance of this study, it’s essential to consider previous research in the field. Studies by Smith et al. [7] and Johnson & White [8] have highlighted the growing importance of cloud computing and the increasing risk posed by DDoS attacks in the cloud. Additionally, works by Amine et al. [9] and Mahmood et al. [10] have explored machine learning-based approaches to address cloud vulnerabilities.

However, these studies also emphasize the need for more accurate and efficient detection mechanisms, particularly for application-layer DDoS attacks. This study aligns with these concerns and aims to deliver a robust solution that improves accuracy while maintaining efficiency.

In summary, this research addresses the critical challenges posed by DDoS attacks in cloud computing. By introducing the Bayesian-based CNN model and data fusion enhancements, it endeavors to provide a groundbreaking solution that not only advances state-of-the-art but also enhances the security and reliability of cloud-based services.

## 2. Research Questions

To address these challenges, this study aims to answer the following research questions:

- How effective is the proposed Bayesian-based CNN (BaysCNN) model in detecting DDoS attacks in cloud computing environments?
- To what extent does the incorporation of the Data Fusion BaysFusCNN approach improve the accuracy, reliability, and key performance metrics (e.g., accuracy, false positive rates) of DDoS detection compared to the BaysCNN model alone and existing methods?
- How do the proposed models compare to existing DDoS detection methods in terms of accuracy and efficiency?

These research questions guide our investigation into the development and evaluation of novel machine learning approaches for DDoS cloud detection, shedding light on the potential improvements and advancements in cloud security.

## 3. Literature Survey

In this section, we review key studies that have paved the way for our novel DDoS detection approach. We compare their methods and limitations, setting the stage for our BaysCNN model’s contributions. Table 1 provides a comparative analysis of these previous studies.

One of the seminal works in this domain was conducted by Bouzida et al. (2006) [11], who employed decision trees (DT) and neural networks (RNN) as supervised learning methods to detect DDoS attacks using the KDD dataset. While their approach showed promise, it struggled to detect new and evolving attack patterns. In contrast, our BaysCNN model, comprising 19 layers of analysis, exhibits a remarkable average accuracy rate of 99.66% across 13 multi-class attacks, showcasing significant advancements in detection capabilities.

Sabeel et al. (2019) [12] presented machine learning models, DNN and LSTM, for predicting DoS and DDoS attacks using the CICIDS2017 dataset. Their models demonstrated high accuracy but faced challenges in real-time detection. In contrast, our BaysCNN model offers superior accuracy, bolstering real-time detection capabilities, and is further enhanced through the Data Fusion BaysFusCNN approach, achieving an average accuracy of 99.78% across 13 multi-class attacks. This fusion model employs Bayesian techniques to estimate uncertainties and combines features from multiple sources, offering a holistic solution for DDoS cloud detection.

Amma and Subramanian (2019) [13] introduced the Vector Convolutional Deep Feature Learning (VCDeepFL) technique for DDoS attack identification, outperforming existing methods in terms of accuracy and detection rates. However, their reliance on an outdated dataset and the absence of trials for identifying unknown assaults present limitations. Our proposed BaysCNN model addresses these concerns and significantly enhances detection accuracy.

Li and Lu (2019) [14] combined LSTM and the Bayes technique to develop the LSTM-BA model, excelling in the F1 Score and accuracy compared to other models. Nevertheless, its slower attack detection speed limits its suitability for real-time scenarios. Our BaysCNN model offers a balance between detection speed and accuracy, providing robust real-time capabilities.

In their work, Gaikwad et al. (2015) [15] implemented the Bagging Ensemble framework for IDS using Partial Decision Trees. While their approach demonstrated high classification accuracy, it suffered from extended model construction times. Our BaysCNN model offers improved performance without sacrificing efficiency.

Roopak et al. (2020) [16] employed multi-objective optimization and CNN-LSTM fusion for attack classification, obtaining promising results. However, their method lacks information on attack characteristics. Our approach leverages Bayesian methods and data fusion, enhancing both accuracy and interpretability.

Marir et al. (2018) [17] introduced a hybrid DBN-SVM model for anomaly detection, delivering strong results but at the cost of increased complexity. Our BaysCNN model offers comparable performance with improved efficiency.

Youseef Alotaibi et al. (2024) [18] introduced an innovative approach titled “Inverse chi-square-based flamingo search optimization with machine learning-based security solution for Internet of Things (IoT) edge devices”. Their study is centered around threat recognition in IoT edge devices, utilizing the ICSFSO-ML technique. This technique integrates the ICSFSO algorithm, SBiLSTM model, and AOA for hyperparameter selection, resulting in an impressive maximum accuracy of 98.25%.

In contrast, our work extends beyond the focus on IoT edge device security. The proposed BaysCNN and BaysFusCNN models broaden the application to cloud-based Distributed Denial of Service (DDoS) detection. Both models make significant contributions to the field, with Alotaibi et al.’s ICSFSO-ML technique addressing specific challenges in IoT security. In comparison, our models aim to enhance the overall reliability and scalability of intrusion detection systems in cloud computing environments.

In summary, the proposed BaysCNN and BaysFusCNN models present a significant leap forward in DDoS cloud detection within the context of cloud computing. These models leverage Bayesian methods and feature fusion to achieve high accuracy, real-time capabilities, and improved efficiency, addressing the limitations of prior studies. This work not only enhances the reliability and scalability of IDS in cloud computing environments but also empowers organizations to proactively mitigate security risks and safeguard against cyber-attacks. 

## 4. Methodology

In this section, we elaborate on our methodology for detecting and classifying Distributed Denial of Service (DDoS) attacks in the CICDDOS2019 dataset. Our approach combines Bayesian Machine Learning (ML) and Convolutional Neural Networks (CNN) within a Bayesian Convolutional Neural Network (BaysCNN) model. The BaysCNN model employs a probabilistic approach, which considers the probabilities and uncertainties associated with network traffic patterns, while the CNN component enables the analysis of complex and extensive datasets [19,20].

To further enhance the effectiveness of this detection system, we introduce the Data Fusion BaysFusCNN approach, which leverages Bayesian methods to estimate uncertainty and fuse extracted features from multiple sources. The process begins with data collection, cleaning, and preprocessing. Relevant features are then extracted, and dimensionality reduction is performed using Principal Component Analysis (PCA). Subsequently, we developed and trained the BaysCNN classifier, followed by model validation and evaluation using appropriate metrics such as accuracy, precision, recall, and F1 Score. Finally, we save the details of the trained model. 

Incorporating the Data Fusion BaysFusCNN approach involves estimating uncertainty and fusing features extracted from multiple data sources. The new model is developed and trained, and its results are compared with those of the BaysCNN model and other similar models. In summary, our proposed approach revolves around the utilization of a Bayesian Convolutional Neural Network (BaysCNN) model for detecting DDoS attacks. The introduction of the Data Fusion BaysFusCNN approach not only allows us to estimate uncertainty but also significantly enhances the accuracy of the detection system.

### 4.1. Preprocessing

In our research, we conducted a thorough pre-processing of the CICDDoS2019 dataset to extract and refine relevant features essential for our model’s performance. This pre-processing phase was a critical step to ensure the accuracy and reliability of our results. It involved several key phases, including data gathering, sampling, data cleaning, feature selection, label encoding, normalization, and principal component analysis (PCA). Each of these steps was meticulously executed to enhance the quality of the dataset and prepare it for subsequent model development.

To accomplish this pre-processing, we employed Python version 3.7 code sourced from [21,22]. This Python-based approach efficiently reduced the dataset’s dimensionality while retaining crucial features. By leveraging Python for pre-processing and MATLAB for model development, we harnessed the strengths of both platforms to create an efficient and effective solution for detecting and classifying Distributed Denial of Service (DDoS) attacks in cloud computing environments.

**Figure 1:**
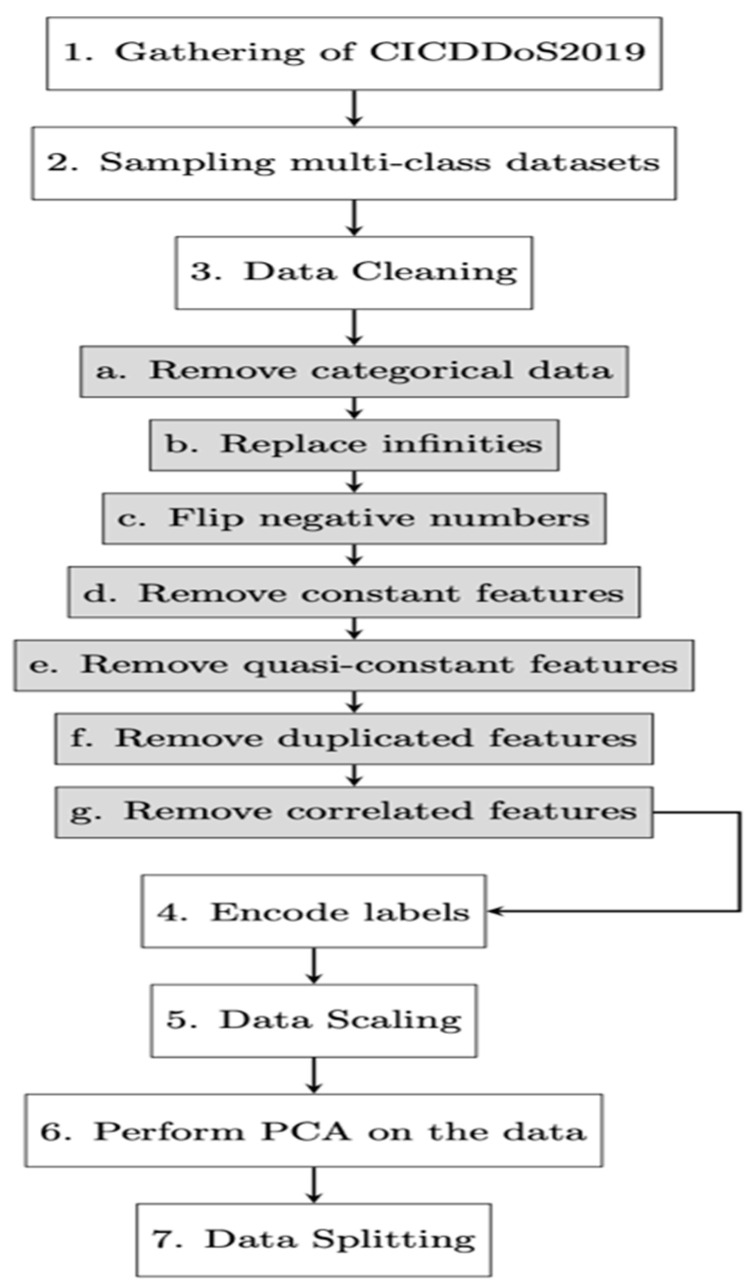
Preprocessing of The CICDDoS2019 Dataset Phases.

Our proposed model architecture, combined with these pre-processing techniques, carries significant implications for enhancing cloud computing system security. This versatile model can be deployed for various applications, including intrusion detection, network traffic analysis, attack mitigation, and prevention. The visual representation of our pre-processing steps is provided in Figure 1, illustrating the sequential flow from data gathering to data splitting.

#### 4.1.1. Gathering of Data: Canadian Institute for Cyber-Security Datasets

The CICDDoS2019 dataset is a comprehensive collection of network traffic data that captures both benign and contemporary DDoS (Distributed Denial of Service) attacks [19,20]. The dataset is presented in CSV file format and incorporates the outcomes of network traffic analysis using CICFlowMeter-V3. The flows in the dataset are labeled based on various attributes, including timestamp, source and destination IPs, source and destination ports, protocols, and the occurrence of attacks. One notable feature of this dataset is the inclusion of various modern reflective DDoS attacks, such as PortMap, NetBIOS, LDAP, MSSQL, UDP, UDP-Lag, SYN, NTP, DNS, and SNMP. These attacks were intentionally executed during the dataset creation period. The dataset is divided into a training day and a testing day, each featuring different sets of DDoS attacks. The training day involved the execution of 12 DDoS attacks, including NTP, DNS, LDAP, MSSQL, NetBIOS, SNMP, SSDP, UDP, UDP-Lag, WebDDoS, SYN, and TFTP. The testing day included seven attacks, namely PortScan, NetBIOS, LDAP, MSSQL, UDP, UDP-Lag, and SYN. Notably, WebDDoS had low traffic volume, and PortScan was only executed on the testing day.

The dataset was developed by the Canadian Institute for Cybersecurity (CIC) and is designed to be a valuable resource for cybersecurity research and analysis. It encompasses various features crucial for understanding and analyzing network traffic, including source and destination IP addresses, port numbers, protocol information, timestamps, packet sizes, and attack scenarios. The dataset is particularly useful for benchmarking, as it provides pre-classified attack labels. In total, the CICDDoS2019 dataset comprises 88 attack scenarios, each generated from different attack types conducted on victim servers hosted on platforms like Amazon Web Services (AWS) and Microsoft Azure [23]. The class distribution of the dataset is detailed in Table 2, offering insights into the diversity and prevalence of various attack scenarios. 

#### 4.1.2. Sampling Multi-Class Datasets

Due to class imbalance, sampling techniques are employed to balance the dataset and improve classification performance. The Pielou Index under-sampling method [24] is used to address this issue. The following steps are followed to calculate the Pielou Index [24]:

1. Obtain the class distribution of the original dataset.
2. Calculate the Shannon Diversity Index (*H*) using the formula:

(1) $$H=-\sum \left(pi\times \log_{2}pi\right)$$

where *pi* represents the proportion of instances in each class.

3. Calculate the maximum possible diversity (*Hmax*) using the formula:

(2) $$Hmax=\log_{2}N$$

where *N* is the total number of instances.

4. Calculate the Pielou Index (*J*) by dividing *H* by *Hmax*.

(3) $$J=\;\frac{H}{Hmax}\;$$

**Figure 2:**
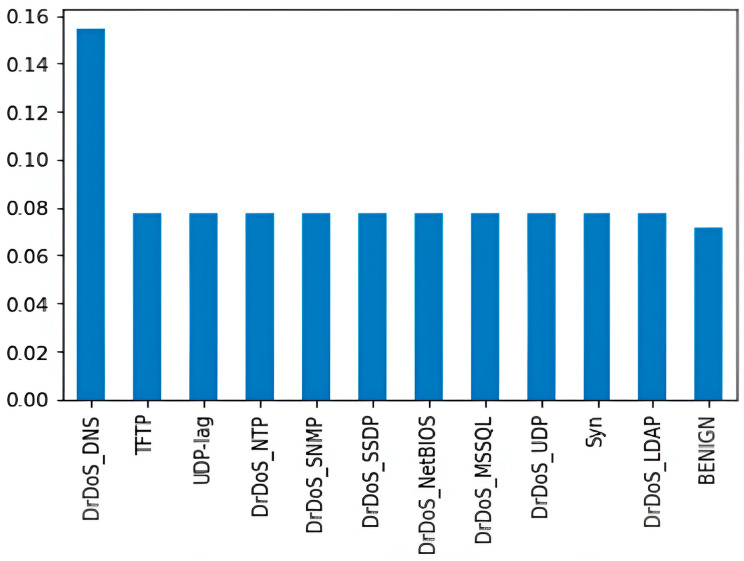
CICDDOS 2019 Dataset Class Distribution After Sampling Chart.

The ‘WebDDoS’ class, with a significantly smaller sample size, is removed. Table 3 and Figure 2 illustrate the updated class distribution after sampling.

#### 4.1.3. Data Cleaning

##### Removing Categorical Data

Columns with the ‘object’ data type, representing categorical features, are excluded from the dataset. These columns include ‘Flow ID’, ‘Source IP’, ‘Source Port’, ‘Destination IP’, ‘Destination Port’, ‘Protocol’, ‘Timestamp’, ‘SimillarHTTP’, and ‘Inbound’, reducing the column count from 88 to 78.

##### Replace Infinities

Infinite values in the dataset are replaced with NaN (Not a Number) to ensure proper data processing. NaN values are then replaced by the maximum value of the respective feature.

##### Flipping Negative Numbers

Negative values are converted to their absolute values to ensure non-negativity, a requirement for the machine learning model.

##### Remove Constant Features

Constant features, those with identical values across all instances, are removed in the pre-processing stage. This step aims to enhance the model’s generalization capability by reducing dimensionality and preventing overfitting. In our dataset, 13 constant columns were identified and subsequently removed, resulting in a reduction of the column count from 78 to 65.

##### Remove Quasi-Constant Features

To further optimize model performance, quasi-constant features, characterized by minimal variation among instances, are eliminated. Features exhibiting variances below a specified threshold (e.g., 0.01) are considered quasi-constant and, as such, are removed from the dataset. In this instance, three quasi-constant columns were identified and excluded, leading to a reduction in the column count to 62.

The removal of constant and quasi-constant features is a crucial pre-processing step, as it ensures that the model focuses on relevant and informative features, contributing to improved efficiency and accuracy in subsequent analyses.

##### Remove Duplicated Features

Duplicate features are removed to eliminate redundant information. After this step, the column count is reduced to 61.

##### Remove Correlated Features

**Figure 3:**
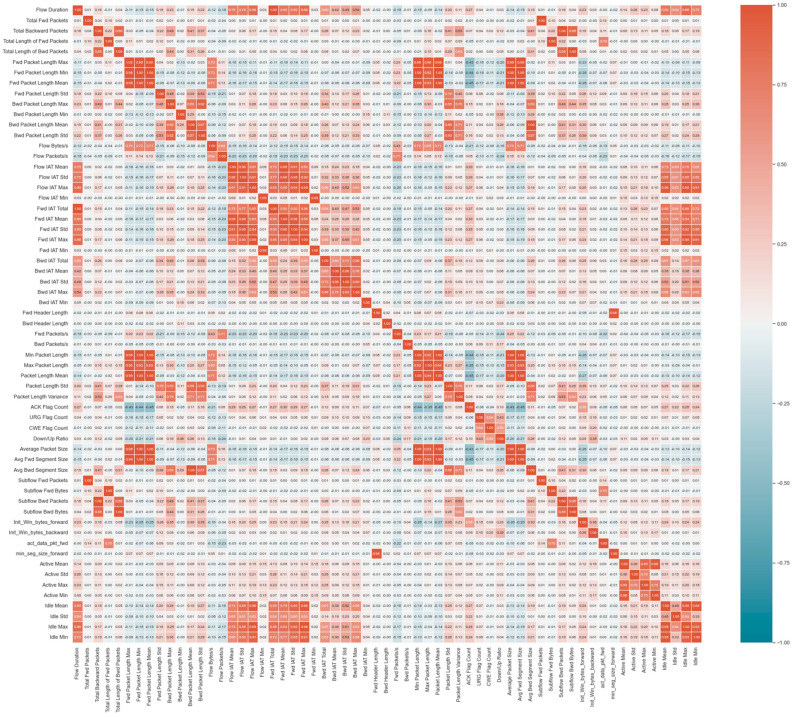
The Heatmap of Correlated Features.

Removing correlated features aimed to further enhance machine learning algorithms by reducing redundancy and multicollinearity. Figure 3 illustrates the heatmap of correlated features. To execute this removal, the dataset was loaded as a Pandas data frame, and the correlation matrix between all feature pairs was calculated using the Corr () method. The upper triangle of the correlation matrix, excluding the diagonal, was examined to prevent duplicate calculations. Highly correlated columns were identified using correlation thresholds of 0.9, 0.99, and 0.999. A total of 10 correlated groups were detected out of the initial 61 features. These correlated columns were removed from the dataset, ultimately reducing the column count from 61 to 42.

#### 4.1.4. Encode Labels Using One-Hot Encoding

The technique of one-hot encoding involves the conversion of categorical variables into a numeric format that is comprehensible to models. The objective is accomplished through the creation of a binary vector, wherein each distinct category is assigned a unique bit value. In this context, every feature is denoted as a binary vector consisting of 0 s and 1 s. The presence of a one at a particular index inside the vector signifies the membership of the feature in a specific category.

#### 4.1.5. Data Scaling

Normalization is employed to scale the data into a range suitable for different algorithms. Min–max scaling is used to transform data to the range [0, 1] using the following formula:

(4) $$mydata\;=\frac{\;\left(mydata-minData\right)}{\left(maxData-minData\right)}.$$

Equation (4) represents the min-max scaling process, a common technique in data preprocessing to ensure that numerical features are on a similar scale. Let’s break down the components of this equation:

- mydatamydata: This is the feature or variable that we want to scale.
- minDataminData: Represents the minimum value of the feature.
- maxDatamaxData: Represents the maximum value of the feature.

The purpose of this equation is to transform the values of mydata into a normalized range between 0 and 1. The numerator (*mydata − minData*) calculates the range between the minimum and current values and dividing it by (*maxData − minData*) scales this range to fit within [0, 1]. Now, Equation (5) represents an additional transformation applied after the min-max scaling:

(5)              mydata=mydata×2−1 

This equation scales the *mydata* values, which have undergone min-max scaling, to a new range. Let’s break down the components of this equation:

- *mydata*: The feature or variable that has undergone min-max scaling.
- The multiplication by 2 expands the range of the scaled values.
- Subtracting one shifts the range to be centered around 0.

The purpose of this transformation is to map the scaled values from the range [0, 1] to the range [−1, 1]. This adjustment can be beneficial in certain machine learning algorithms that perform better when the data are centered around zero.

Therefore, both Equations (4) and (5) collectively illustrate the process of data scaling using min-max scaling followed by an additional adjustment to center the values around 0. These techniques ensure that the features are appropriately normalized and centered for effective use in various algorithms.

#### 4.1.6. Implementing PCA

**Figure 4:**
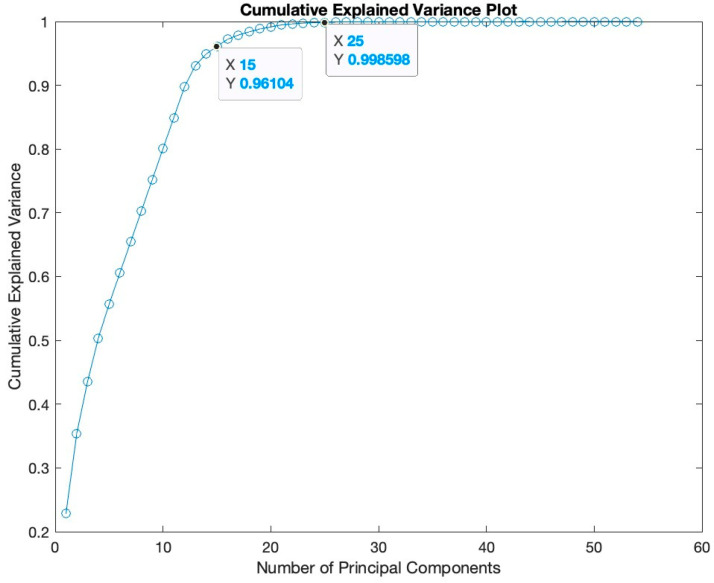
Cumulative Explained Variance Plot.

To enhance computational efficiency and mitigate the risk of overfitting, PCA-based pre-processing techniques were employed in the proposed study. The original 41 features were transformed into a set of uncorrelated principal components using Principal Component Analysis (PCA). The decision to utilize PCA was supported by a comprehensive analysis depicted in the cumulative explained variance plot (refer to Figure 4). This plot illustrates the trade-off between dimensionality reduction and retaining dataset variance. Our aim was to retain at least 95% of the total variance, and based on the cumulative explained variance plot, a specific number of principal components were selected. Notably, the final features for the study include two principal components capturing 35.4% of the variance, four components representing 50.35%, seven components at 65.4%, ten components at 80.05%, 12 components at 89.7%, 19 components at 98%, and 25 components at an impressive 99.8%. This meticulous selection ensures an optimal balance between reducing dimensionality and preserving crucial information in the dataset.

#### 4.1.7. Data Splitting

The dataset is split into training, validation, and test sets for model training, hyperparameter tuning, and evaluation. The split is as follows: 45% for training, 25% for validation, and 30% for testing.

### 4.2. BaysCNN Model

**Figure 5:**
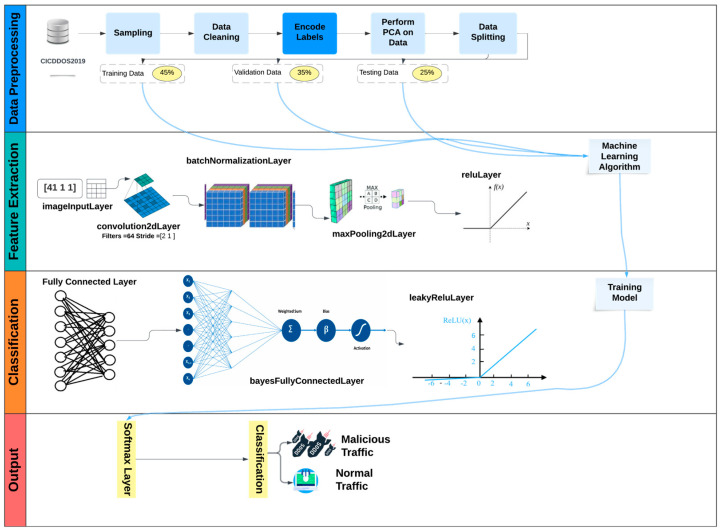
General Structure of BaysCNN Model.

Our methodology commences with the development of the Bayesian Convolutional Neural Network (BaysCNN) model. This model is designed to effectively detect and classify DDoS attacks within cloud computing systems. The BaysCNN model, as illustrated in Figure 5, provides a high-level overview of its structure. This schematic diagram outlines the fundamental components and flow of information within the model.

The BaysCNN model offers several key advantages:

- Complex Pattern Recognition: The combination of Bayesian methods and CNN allows for the accurate identification and prediction of complex data patterns within network traffic data.
- Probabilistic Approach: Bayesian ML introduces a probabilistic perspective, enabling us to assess the uncertainties associated with network traffic patterns. This helps improve the model’s robustness in the presence of noise or variations.

#### 4.2.1. Model Architecture Overview

The BaysCNN model architecture comprises 19 layers, each contributing to the model’s ability to understand and classify network traffic patterns effectively. The architectural flow is as follows:

1. Input Layer: The model begins with an imageInputLayer of dimensions [41 1 1] corresponding to the size of the input data.
2. Convolutional Layers: The input data undergoes convolution via a convolution2dLayer with 64 neurons. This is followed by a batchNormalizationLayer and maxpooling2dLayer. The output of these layers is then passed through a ReLU layer.
3. Fully Connected Layers: Following the convolutional layers, the data flows through a fullyConnectedLayer with 200 neurons and two BayesFullyConnectedLayers with 784 output neurons and 392 output neurons.
4. Intermediate Layers: The architecture includes additional layers, including ReLU, batchNormalizationLayer, and leakyReLU, to further enhance feature extraction and representation.
5. Output Layer: The model concludes with a SoftmaxLayer and a Classification Layer.

#### 4.2.2. Practical Implementation

In this section, we delve into the practical implementation of the BaysCNN and BaysFusCNN models for detecting DDoS attacks within the context of cloud computing environments.

#### 4.2.3. Developing BaysCNN Model

##### The Complete Structure

**Figure 6:**
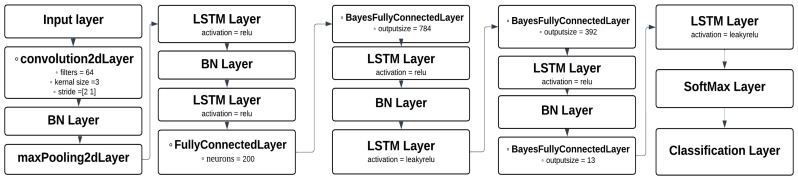
BaysCNN Model Architecture.

The BaysCNN model, a cornerstone of our research, boasts a comprehensive architecture consisting of 19 layers, each meticulously designed to fulfill specific roles within the network flow. Illustrated in Figure 6, this model exemplifies our commitment to robust neural network design optimized for the unique characteristics of our dataset.

The journey through the BaysCNN architecture commences with an imageInputLayer tailored to accommodate input data of dimensions [41 1 1], aligning seamlessly with the size of our input data. This initial layer serves as the entry point for our spectrogram data.

The input data subsequently embarks on a transformative journey. It first encounters a convolution2dLayer, housing 64 neurons, employing a kernel size of 3 × 3 and a stride of 1. This convolutional layer efficiently extracts critical features from the input spectrograms. To ensure stability and expedited convergence, a batchNormalizationLayer follows, regulating the internal activations. Next in line is the maxPooling2dLayer, which strategically reduces spatial dimensions while retaining essential information. This is succeeded by a ReLU layer, facilitating non-linearity in feature extraction.

Continuing this architectural journey, the output of the initial convolutional layers undergoes further processing. A fullyConnectedLayer comprised of 200 neurons provides a robust feature representation, which is subsequently passed to a BayesFullyConnectedLayer equipped with 784 output neurons. This Bayesian layer introduces probabilistic weight distributions into the network, adding an extra layer of expressiveness and uncertainty modeling.

Following the Bayesian layer, a ReLU layer further enhances non-linearity, and another batchNormalizationLayer promotes stable training. The introduction of a leakyReLU layer introduces a controlled degree of non-linearity to capture complex patterns.

The model then encounters another Bayesian layer, this time featuring 392 output neurons, further diversifying its capabilities. A ReLU layer, batchNormalizationLayer, and a final BayesFullyConnectedLayer with 13 output neurons refine and distill the feature representations, culminating in a final leakyReLU layer.

The journey concludes with a SoftMax layer, which provides probability scores for each class, and a classificationLayer for final classification. In the preprocessing stage, Short-Time Fourier Transform (STFT) is applied to the input data, yielding two-dimensional spectrograms. These spectrograms are then fed into the convolutional segment of the network.

#### 4.2.4. Developing BaysFusCNN Architecture

**Figure 7:**
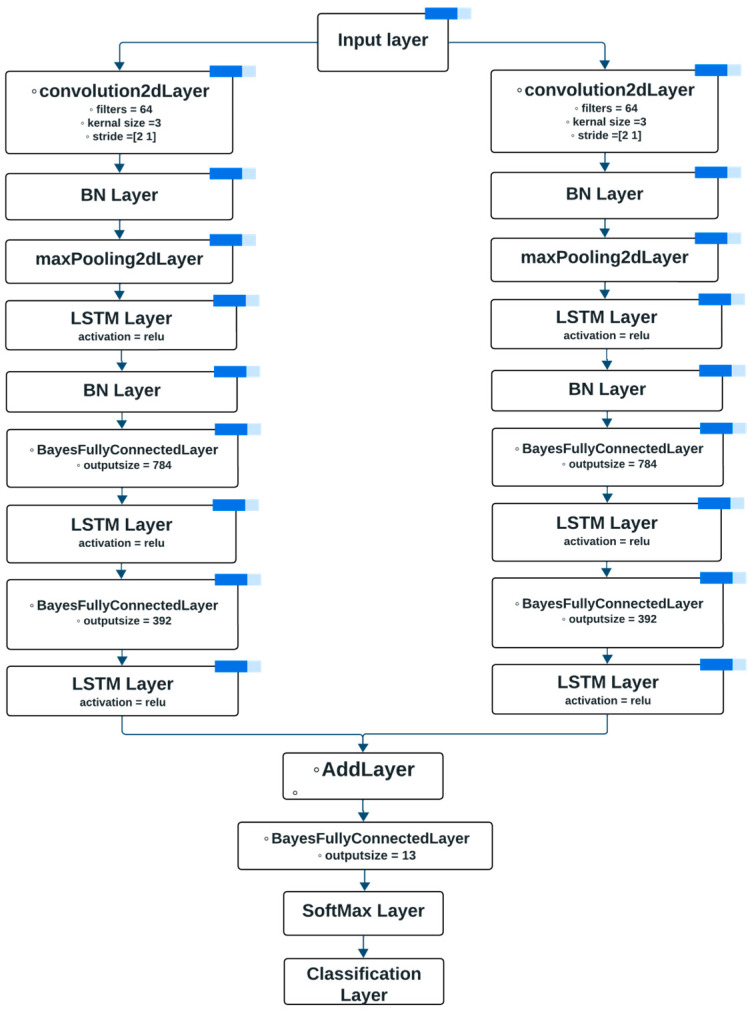
BaysFusCNN Model Architecture.

The BaysFusCNN model, depicted in Figure 7, boasts a comprehensive architecture comprised of 27 layers, with a total of approximately 2.2 million learnable parameters. This section provides a detailed insight into the architecture’s design and specifications.

1. Input Layer: The model’s journey begins with an input layer tailored to accommodate data with dimensions of 41 × 1 × 1, designed to seamlessly integrate with the size of the input data.
2. Initial Convolutional Layer: The input data are then processed through an initial convolutional layer featuring 16 filters, a padding of 1, and a kernel size of 3. This layer efficiently extracts essential features from the input spectrograms.
3. Sequential Processing: The output from the initial convolutional layer undergoes sequential processing, featuring:
  - Batch Normalization Layer: To ensure stability and expedited convergence.
  - Max Pooling Layer: With pooling size [2 1] and a stride of [2 1], effectively reducing spatial dimensions.
  - ReLU Activation Layer: To introduce non-linearity into feature extraction.
4. Bayesian Fully Connected Layers: The output from the sequential processing encounters two Bayesian fully connected layers, with output capacities of 784 and 392, respectively. These layers introduce probabilistic weight distributions into the network, enhancing expressiveness and uncertainty modeling. The model parameters, Sigma1 and Sigma2, are tuned to values of 1 and 0.5, respectively.
5. Intermediate Processing: The output from the initial Bayesian fully connected layer proceeds through:
  - Leaky ReLU Activation Layer: To capture complex patterns.
  - Batch Normalization Layer: Ensuring stable training.
  - Second Bayesian Fully Connected Layer: Further refine and diversify feature representations.
  - Leaky ReLU Activation Layer: Introducing controlled non-linearity.
6. Parallel Branch: Simultaneously, the input data undergoes an alternative branch, mirroring the specifications of the initial branch’s convolutional layer, batch normalization layer, max pooling layer, and ReLU activation layer. Like the initial branch, this branch is also subjected to batch normalization.
7. Concatenation and Output: The outputs from both branches are concatenated, creating a fusion of features. This fused output proceeds through a softmax layer, culminating in the model’s final output.
8. Training and Evaluation: Training the network involves the propagation of training data and predictor data through the network, guided by the options outlined in the ‘options’ variable. Subsequently, the trained network is evaluated using test data to assess its efficacy in classifying input data.

#### 4.2.5. Experimental Setup

In this section, we describe the experimental setup, including the hardware and software used, training options, and evaluation metrics for our deep learning models.

##### Hardware Specifications

We conducted our experiments on a 16-inch MacBook Pro equipped with the following Table 4 hardware components. This hardware configuration provided the necessary computational power and resources for our deep learning experiments.

##### Software Specification

Our experiments were conducted using the following software tools:

- MATLAB: MATLAB version R2023b was utilized for model development, training, and evaluation. It offers a versatile environment for deep learning and data analysis.
- Python 3.7: Python 3.7 was employed for preprocessing the dataset, making use of its rich ecosystem of libraries for data manipulation.

#### Training Options

To train our deep learning models effectively, we carefully selected and configured various training options using MATLAB’s R2023b trainingOptions function. Table 5 provides a detailed list of the training options we employed:

##### Evaluation Metrics

In our classification experiments, we employ several key evaluation metrics to assess the performance of our models:

Evaluation metrics are important measures used to evaluate the performance of machine learning models. In model classification, some common evaluation metrics include accuracy, recall, precision, specificity, and F1 Score.

1. Accuracy

*Accuracy* is a measure of the total number of correct predictions made by the model compared to the total number of samples in the dataset [25]. It is calculated as:

(6) $$Accuracy=\frac{\left(TP+TN\right)}{\left(TP+TN+FP+FN\right)}$$

where *TP* (True Positive) represents the number of correctly predicted positive samples, *TN* (True Negative) represents the number of correctly predicted negative samples, *FP* (False Positive) represents the number of incorrectly predicted positive samples and *FN* (False Negative) represents the number of incorrectly predicted negative samples.

2. Recall (Sensitivity)

*Recall* (also known as sensitivity) is the proportion of true positive samples correctly classified by the model [25]. It is calculated as:

(7) $$Recall=\frac{TP}{\left(TP+FN\right)}$$

3. Precision

*Precision* is the proportion of correctly predicted positive samples among all predicted positive samples [25]. Precision is calculated as:

(8) $$Precision=\frac{TP}{\left(TP+FP\right)}$$

4. Specificity

*Specificity* is the proportion of true negative samples correctly classified by the model [25]. Specificity is calculated as:

(9) $$Specificity=\frac{TN}{\left(TN+FP\right)}\;\;\;\;\;$$

5. Fall-out (False Positive Rate)

*Fall-out*, also known as the False Positive Rate (FPR), is the proportion of true negative samples incorrectly classified by the model [25]. Fall-out can be calculated as follows:

(10) $$Fall-out=\frac{FP}{\left(TN+FP\right)}$$

6. F1 Score

*F*1 *Score* is the harmonic mean between precision and recall, providing an overall measure of the model’s performance [25]. It is calculated as:

(11) $$F1\;Score=2\times \frac{\left(Precision\times Recall\right)}{\left(Precision+Recall\right)}$$

These evaluation metrics are essential to measure the performance of the classification models. They provide valuable insights into how well the model is performing and can guide the model improvement processes.

## 5. Results

In this section, we present the performance and findings of both the BaysCNN and BaysFusCNN models, which have been trained and evaluated on a dataset consisting of various network traffic samples. We discuss the evaluation metrics that provide valuable insights into the models’ accuracy, precision, recall, specificity, and the F1 Score for each class. Analyzing these metrics allows us to gain a comprehensive understanding of the effectiveness of both models in classifying different types of network traffic.

### 5.1. BaysCNN Model Results

In this subsection, we delve into the performance of the BaysCNN model. We provide a detailed analysis of its classification results, highlighting its strengths and areas for improvement. The evaluation metrics, such as accuracy, recall, precision, specificity, F1 Score, and the overall average accuracy of approximately 99.66%, reveal the remarkable performance of the BaysCNN model.

Accuracy: The accuracy of the BaysCNN model demonstrates its ability to classify network traffic accurately, ranging from 99.43% for the Portmap class to an impressive 99.84% for the DrDoS_DNS class, as detailed in Table 6. These figures signify the model’s effectiveness in distinguishing between different traffic types, emphasizing its robust performance.

Recall (TP Recall): The recall rate, ranging from 95.01% for the DrDoS_LDAP class to 99.67% for the DrDoS_DNS class, reflects the BaysCNN model’s capability to correctly identify positive samples within each class. The model exhibits a high recall, signifying its strength in capturing relevant instances while minimizing false negatives.

Precision (PP Precision): Precision scores vary across different classes, ranging from 97.09% for the Portmap class to a perfect 99.20% for the DrDoS_DNS class. High precision values highlight the model’s accuracy in identifying positive samples and reducing false positives, ensuring reliable classification.

Specificity (AN Specificity): The model’s specificity ranges from 99.78% for the DrDoS_UDP, Portmap, and UDP-lag classes to 99.89% for the DrDoS_LDAP class, indicating its effectiveness in classifying negative samples with minimal false positives. This metric underscores the model’s ability to distinguish non-relevant instances from target traffic classes.

False Positive Recall (FP Recall): FP Recall varies across classes, ranging from approximately 0.11% for the DrDoS_LDAP class to around 0.22% for the DrDoS_UDP, Portmap, and UDP-lag classes. These values quantify the rate of false positives in the model’s predictions, emphasizing its capacity to avoid misclassifying negative samples as positive.

F1 Score: The F1 Score, balancing precision and recall, ranges from 96.06% for the Portmap class to 99.43% for the DrDoS_DNS class. This measurement demonstrates the model’s ability to achieve high levels of both precision and recall simultaneously, ensuring accurate classifications across various traffic types.

Average Performance Metrics: Considering the average performance metrics across all classes, the BaysCNN model maintains an average accuracy of approximately 99.66%, an average recall of 97.66%, an average precision of 97.69%, an average AN specificity of 99.82%, and an average F1 Score of 97.66%. These averages provide a comprehensive overview of the model’s overall performance in classifying diverse network traffic samples.

In conclusion, the evaluation metrics and average performance showcase the exceptional performance of the BaysCNN model in classifying network traffic. Its high accuracy, recall, precision, specificity, and F1 Score underscore its effectiveness in distinguishing different traffic types with precision and reliability.

#### 5.1.1. Confusion Matrix Results of BaysCNN Model

**Figure 8:**
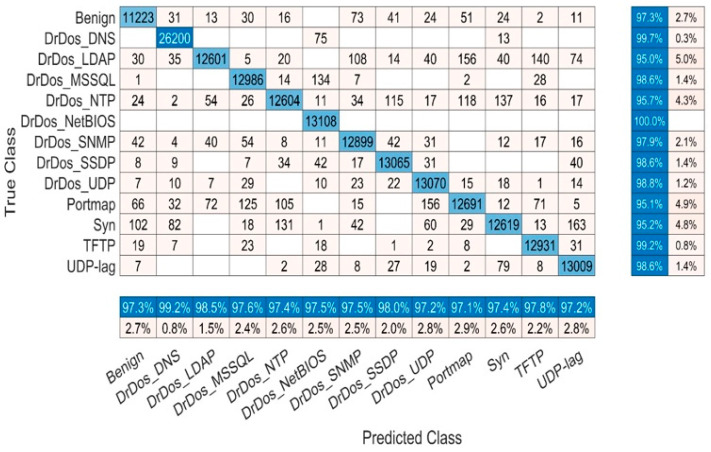
BaysCNN Model Confusion Matrix Chart.

The confusion matrix is a table often used to evaluate the performance of a classification model. It displays the number of correct and incorrect predictions made by the model for each class. Rows represent true labels, and columns represent predicted labels. In the case of the BaysCNN model, the confusion matrix (Figure 8) is a 13 × 13 table, where each row and column correspond to one of the 13 classes. The numbers within the matrix indicate the instances classified into each class. Table 7 presents a detailed analysis of the model’s class-wise performance. Let’s decipher its key elements:

- True Positives (TP): These values signify instances correctly classified into their respective classes. In Class 1, 11,223 true positives imply precise recognition of Class 1 instances.
- False Negatives (FN): FN represents instances of a specific class incorrectly assigned to other classes. In Class 1, 316 false negatives indicate misclassified instances that should belong to Class 1.
- False Positives (FP): FP denotes instances inaccurately classified into a particular class when they do not belong. Class 1, for example, records 306 false positives, signifying instances wrongly labeled as Class 1.

Now, let’s explore the implications:

- High TP: A high count of true positives demonstrates effective recognition of specific class instances.
- Low FN: Few false negatives indicate infrequent misclassification of class instances as something else.
- Low FP: A minimal number of false positives showcases model precision by reducing the misclassification of non-members into a class.

A comprehensive analysis of the confusion matrix identifies patterns and areas for model performance enhancement. Notable results include Class 6 with 13,108 true positives and zero false negatives, highlighting the model’s exceptional ability to classify Class 6 accurately. Class 2 records 26,200 true positives against 88 false negatives, maintaining just 212 false positives, underlining precision and accuracy. These insights inform data-driven improvements to boost the BaysCNN model’s effectiveness and classification accuracy.

#### 5.1.2. Rho Weight Distribution of BaysCNN Model

The BaysCNN model leverages Bayesian principles to elevate its accuracy and reliability [26]. At its core lies the concept of “rho weights”, which assumes a central role in characterizing weight parameter uncertainty [26,27].

In the realm of Bayesian neural networks, weight parameters are treated as probability distributions rather than fixed values [26]. This approach allows the model to express not just point estimates but also the degree of uncertainty in its predictions, a valuable feature for intricate tasks like DDoS attack detection [26].

The rho weight distribution serves as a vital tool during training, helping evaluate the model’s alignment with actual labels [26]. It quantifies the model’s confidence in its predictions [26].

The mathematical foundation of the rho weight distribution adheres to Bayesian principles and is instrumental in estimating uncertainty [26]. Within the BaysCNN model, the rho weights model shows the spread and variability in weight values, characterized by Sigma1 and Sigma2 as prior distribution variances [26,27,28].

Estimating the rho weight distribution involves complex probabilistic calculations, detailed in the provided custom layer code [29]. It constructs a mixture distribution of Gaussian distributions with means at zero and variances determined by Sigma1 and Sigma2, reflecting prior weight uncertainty [29,30].

**Figure 9:**
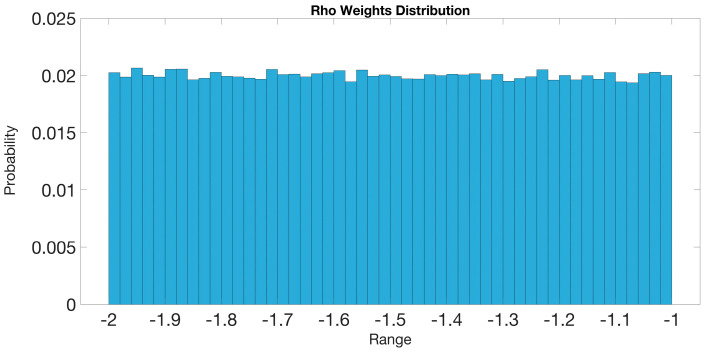
Rho Weights Distribution for BaysCNN Model.

Figure 9 graphically illustrates the rho weight distribution, displaying distributions across multiple training iterations. Each blue line represents a distribution for a specific training iteration, while the black line indicates the average of these distributions. The histogram’s *x*-axis spans weight values from −2 to −1, and the *y*-axis indicates the probability or normalized frequency of occurrence.

Analyzing this graph reveals insights into the BaysCNN model’s behavior. A concentration of bars around a central weight value, approximately 0.02, suggests a significant emphasis on features within this range when making DDoS attack predictions. The uniform distribution around this central value implies consistency in weight assignments across different features.

In summary, the rho weight distribution within the BaysCNN model is a pivotal element of its probabilistic framework. It enables informed predictions while providing a measure of prediction reliability, guided by Bayesian principles and mathematical foundations, enhancing its effectiveness in intricate tasks like DDoS attack detection.

#### 5.1.3. Mean Weight Distribution of BaysCNN Model

**Figure 10:**
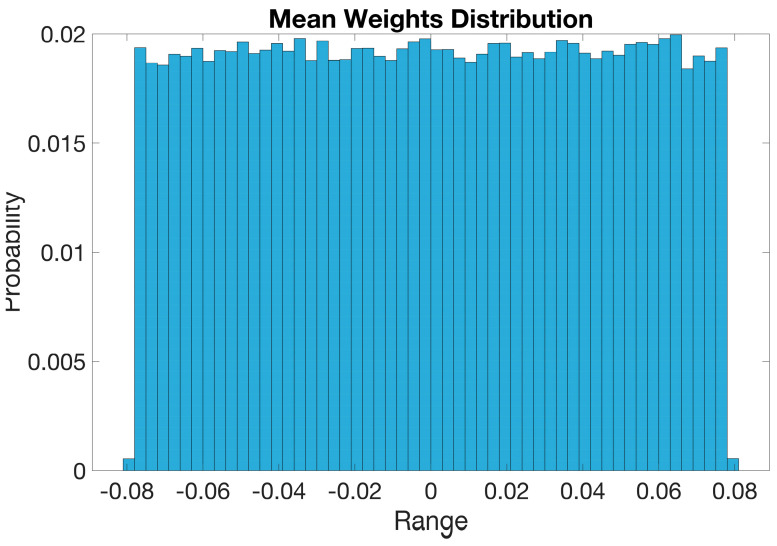
Mean Weights Distribution for BaysCNN Model.

Figure 10 displays the mean weight distribution of a Conv2D layer in the BaysCNN model initialized with Glorot initialization. The histogram divides weights into bins, with the *x*-axis representing weight values and the *y*-axis representing the probability or percentage of weights in each bin. The histogram reveals weight values ranging from −0.08 to 0.08, indicating proper initialization with Glorot. Weights are uniformly distributed across the layer, with a mean weight close to zero. Notably, the graph shows a narrower range of mean weights, approximately −0.08 to 0.08, indicating their prevalence and significance in the model’s predictions. However, two outliers at the extremes suggest the presence of exceptional, low-occurring weights with potential impact on model performance.

#### 5.1.4. Training Progress of BaysCNN Model

**Figure 11:**
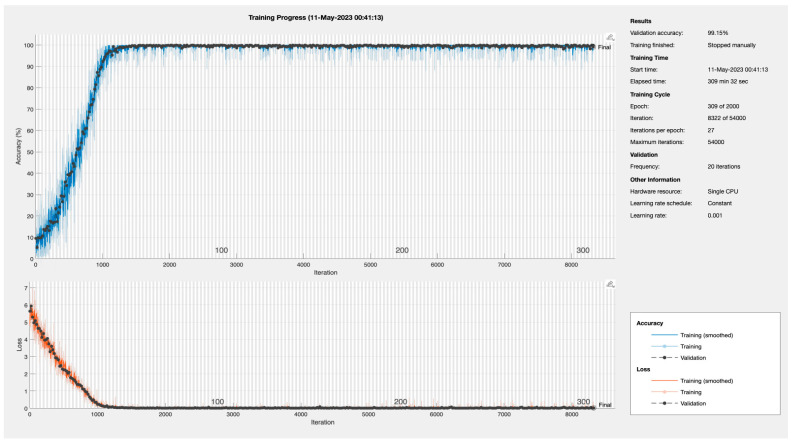
Training Progress for BaysCNN Model.

Figure 11 illustrates the training progress of the BaysCNN machine learning model. Training accuracy steadily increases from 5% to 99–100% over time, indicating improved accuracy in predictions. The model reaches around 99% training accuracy by the 56th epoch. Validation accuracy, measuring performance on a separate dataset, reaches 99.15%, showcasing the model’s effectiveness on unseen data. Simultaneously, the loss, representing the difference between predicted and actual values, decreases from 6.0 to 0, signifying improved prediction accuracy.

These findings demonstrate the significant improvements in accuracy achieved during training, with the model performing exceptionally well with a validation accuracy of 99.15%, validating its effectiveness.

#### 5.1.5. Training Accuracy of BaysCNN Model

**Figure 12:**
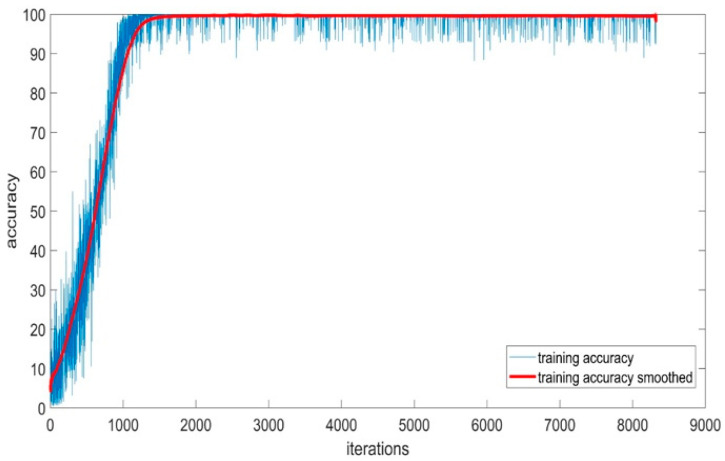
Training Accuracy for BaysCNN Model.

**Figure 13:**
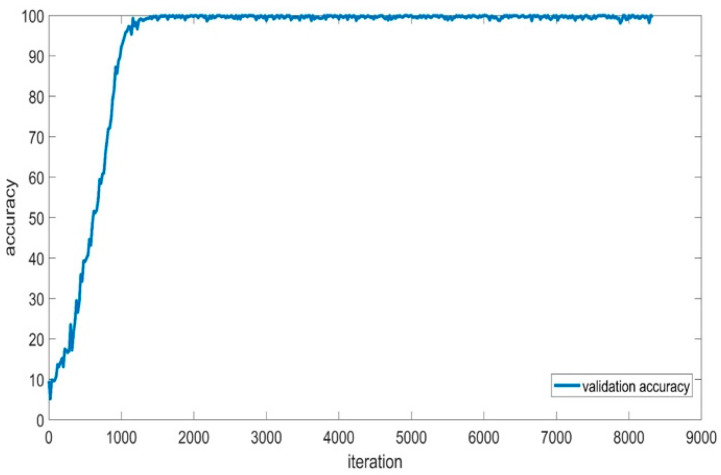
Validation Accuracy for BaysCNN Model.

Figure 12 depicts the training accuracy and smoothed training accuracy of the BaysCNN model over 309 epochs, with each epoch consisting of 27 iterations. The blue line represents training accuracy, gradually increasing over training, indicating the model’s ability to make accurate predictions. The red line, representing smoothed training accuracy, demonstrates a gradual increase, indicating the model’s ability to generalize well to new data. Comparison with validation accuracy in Figure 13 reveals consistently higher validation accuracy, indicating successful generalization and high accuracy on new, unseen data. The model’s exceptional validation accuracy of 99.15% underscores its capability to classify new data.

#### 5.1.6. Training Loss of BaysCNN Model

**Figure 14:**
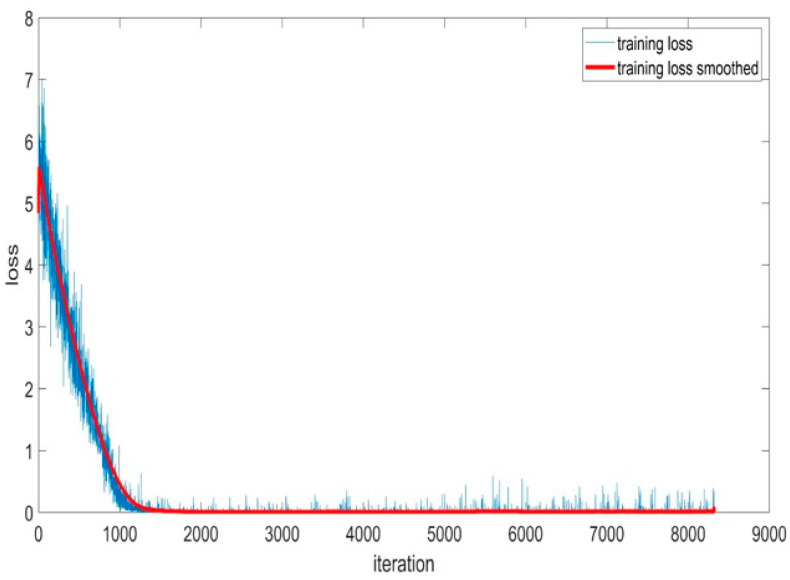
Training Loss Accuracy for BaysCNN Model.

Figure 14 illustrates the training loss of the BaysCNN model throughout 309 training epochs. In this graph, the *y*-axis represents the training loss value, while the *x*-axis represents the number of training iterations.

The blue curve in the graph represents the BaysCNN model’s training loss across multiple epochs, with each epoch consisting of 27 iterations. Concurrently, the red curve depicts a smoothed version of the training loss curve achieved by applying a moving average window of size 20. This smoothing technique provides a clearer view of the loss trend by averaging 20 training loss values centered around each point.

Training loss quantifies the disparity between the model’s predicted outputs and the actual outputs for the training data. During the initial 50 epochs, the training loss experiences a rapid decline, indicating significant improvements in predictive accuracy. Beyond 50 epochs, while the loss continues to decrease, the rate of reduction slows down. The presence of some noise in the training loss curve can be attributed to the stochastic nature of the optimization algorithm employed in model training. The application of a training loss smoother minimizes noise, facilitating the monitoring of the model’s progress.

**Figure 15:**
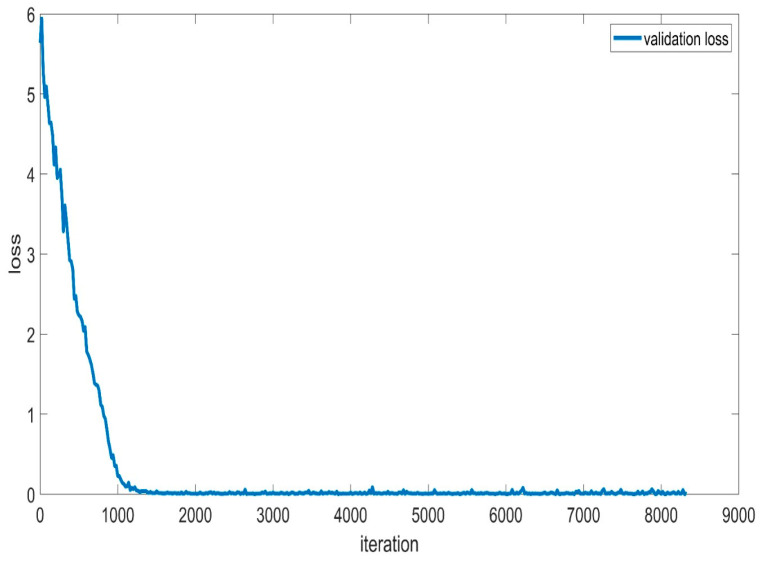
Validation Loss Accuracy for BaysCNN Model.

Additionally, Figure 15 presents the validation loss accuracy, which assesses the BaysCNN model’s performance on a separate validation dataset. Notably, the validation loss accuracy consistently remains lower than the training loss accuracy, suggesting that the model avoids overfitting and generalizes effectively to new data. Furthermore, the validation loss accuracy exhibits gradual improvement over time, signifying enhanced prediction accuracy on unseen data.

The narrowing gap between training loss accuracy and validation loss accuracy further emphasizes the model’s capacity to enhance accuracy while mitigating overfitting.

### 5.2. Results of the BaysFusCNN Model

Table 8 presents a summary of evaluation metrics for the BaysFusCNN model across different traffic classes. These metrics are vital indicators of the model’s classification performance, highlighting its precision in categorizing network traffic. Each row in the table corresponds to a specific class, and the associated metrics are computed for that class.

The BaysFusCNN model achieved remarkable validation and testing accuracy results, averaging approximately 99.79%. Class-specific accuracy ranges from 99.65% to 99.87%, showcasing the model’s proficiency in classifying diverse network traffic samples. Key evaluation metrics include:

- True Positive (TP) Recall: Demonstrates the model’s effectiveness in correctly identifying positive samples within each class. The BaysFusCNN model maintains an average TP Recall of approximately 98.55%, ranging from 97.30% to 99.61% across classes.
- Precision (PP): Measures the model’s accuracy in identifying positive samples while minimizing false positives. The model exhibits an average Precision of approximately 98.57%, with class-specific values ranging from 97.53% to 99.17%.
- Specificity (AN): Evaluates the model’s ability to correctly classify negative samples within each class. The BaysFusCNN model demonstrates an average specificity of 99.88%, varying from 99.81% to 99.92% among classes.
- False Positive Recall (FP Recall): Reflects the rate of false positive predictions by the model. The BaysFusCNN model maintains low FP Recall values, ranging from approximately 0.08% to 0.19% across classes.
- F1 Score: Offers a balanced measure of precision and recall, indicating the model’s overall performance. The average F1 Score stands at 98.56%, with class-specific values spanning from 98.32% to 99.39%.
- Average Performance Metrics: Across all classes, the BaysFusCNN model achieves an average accuracy of approximately 99.79%, an average TP Recall of 98.55%, an average PP Precision of 98.57%, an average Specificity of 99.88%, an average FP Recall of 0.12%, and an average F1 Score of 98.56%. These averages provide a comprehensive overview of the model’s proficiency in classifying diverse network traffic samples.

In conclusion, these evaluation metrics reaffirm the exceptional performance of the BaysFusCNN model in effectively categorizing various network traffic types. Its consistently high accuracy, recall, precision, specificity, and F1 Score values across different classes underscore its remarkable ability to distinguish between various network traffic categories.

#### 5.2.1. Confusion Matrix Results of BaysFusCNN

**Figure 16:**
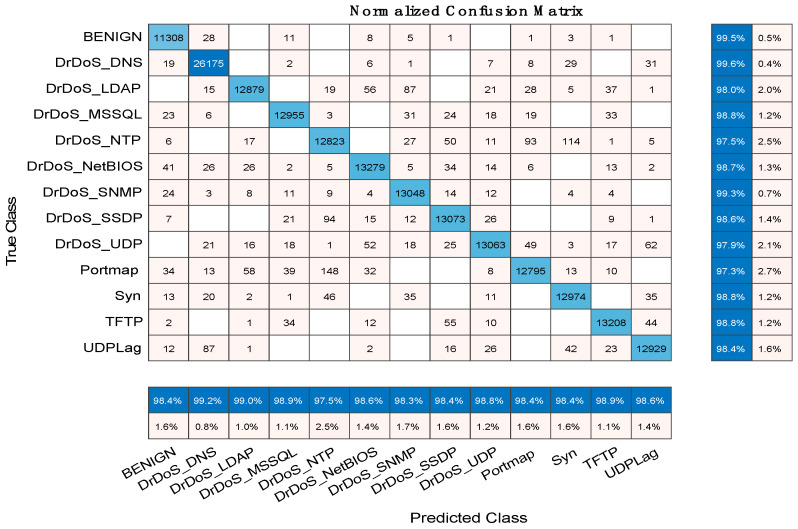
BaysFusCNN Model Confusion Matrix Chart.

The confusion matrix for the BaysFusCNN model, shown in Figure 16 and detailed in Table 9, is a 13 × 13 matrix where each row represents predicted classes, and each column represents actual classes. Interpreting the Confusion Matrix:

- True Positives (TP): These values indicate instances correctly classified into their respective classes. For example, in Class 1, there were 11,308 instances correctly predicted.
- False Negatives (FN): FN values represent instances that belonged to a specific class but were incorrectly classified as something else. In Class 1, there were 58 instances incorrectly classified.
- False Positives (FP): FP values signify instances wrongly classified into a specific class instead of their true class. For instance, in Class 1, there were 181 instances incorrectly predicted.

#### 5.2.2. Training Progress of BaysFusCNN Model

**Figure 17:**
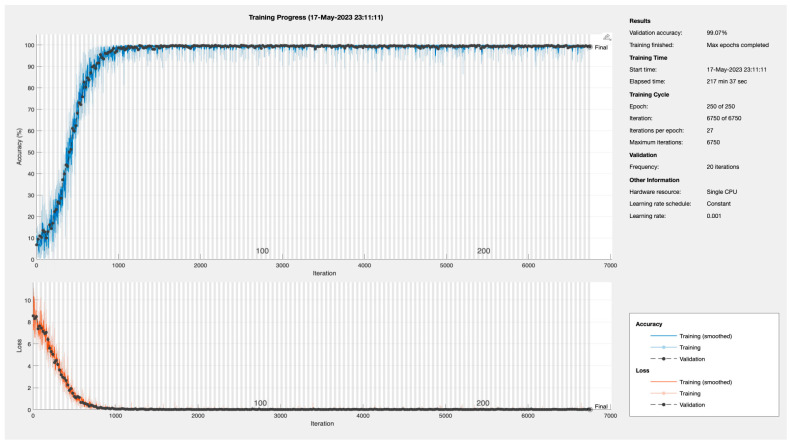
BaysFusCNN Model Training Progress and Validation Accuracy Results.

Figure 17 illustrates the training progress of the BaysFusCNN model across 250 epochs, with each epoch comprising 27 iterations. The *x*-axis denotes the number of epochs (from 1 to 250), while the *y*-axis represents accuracy percentages (ranging from 0% to 100%).

At the outset (epoch 1), the model’s accuracy is approximately 5%. However, with continued learning from the data, accuracy steadily climbs. By the 40th epoch, the accuracy reaches approximately 99%, indicating the model’s ability to make highly accurate predictions on the training data.

The declining loss curve in the figure reflects the model’s improvement over time. The loss represents the difference between predicted and true values. As training progresses, the loss consistently decreases, approaching zero by the training’s end. This reduction signifies the model’s increasing accuracy and alignment with actual values.

Notably, the validation accuracy closely mirrors the training accuracy, reaching 99.07%. This alignment indicates the BaysFusCNN model’s strong generalization capability, maintaining high accuracy even on unseen data.

#### 5.2.3. Training Accuracy of BaysFusCNN Model

**Figure 18:**
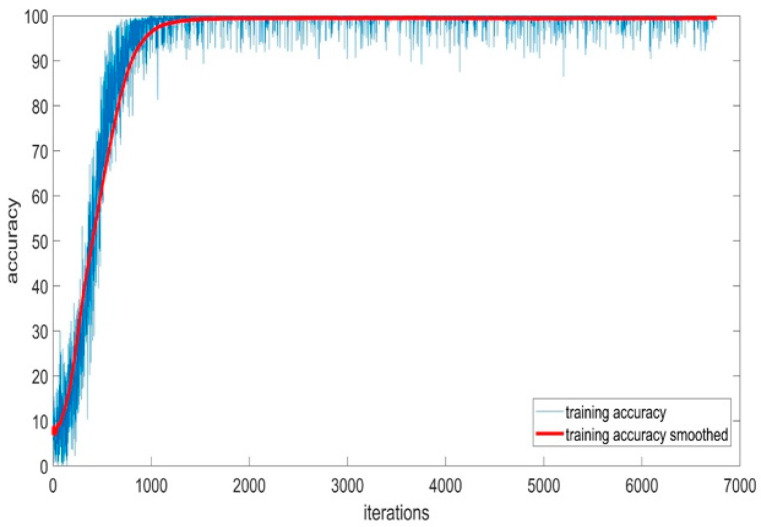
Training Accuracy for BaysFusCNN Model.

Figure 18 displays the training accuracy and smoothed training accuracy of the BaysFusCNN model during 250 training epochs. Each epoch comprises 27 iterations, and the model uses a learning rate of 0.001.

The *x*-axis represents the number of epochs, while the *y*-axis indicates accuracy percentages (ranging from 0 to 100). The blue line represents training accuracy, which steadily increases throughout the training process. This signifies the model’s learning progress and improved accuracy in predicting training data.

The red line, representing smoothed training accuracy, initially shows slight fluctuations over the first epochs, gradually evolving until around epoch 200. From that point onwards, it continues to rise steadily, reflecting consistent improvements while reducing fluctuations.

**Figure 19:**
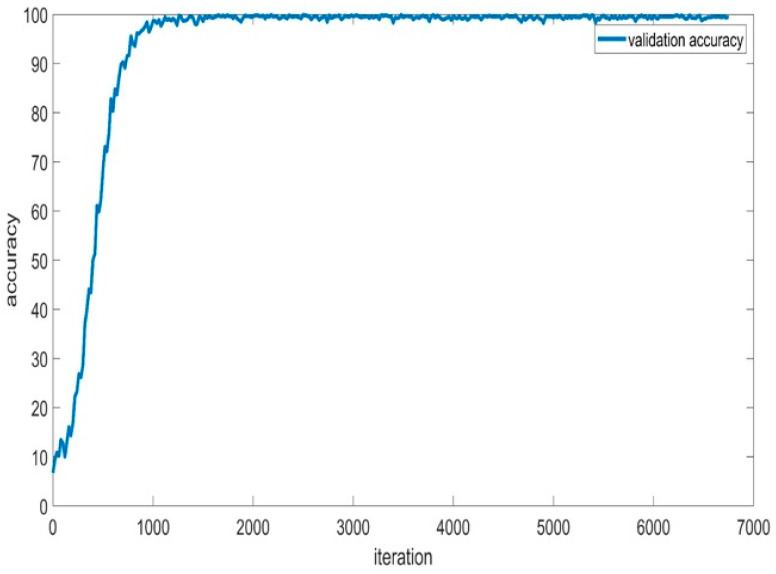
Validation Accuracy for BaysFusCNN Model.

Figure 19 illustrates validation accuracy, which is consistently higher than training accuracy and steadily increases throughout training. The final validation accuracy of 99.07% demonstrates the model’s high accuracy in classifying new data. The alignment between training and validation accuracy suggests that the model has learned data patterns without overfitting, indicating strong generalization to unseen data.

#### 5.2.4. Training Loss of BaysFusCNN Model

**Figure 20:**
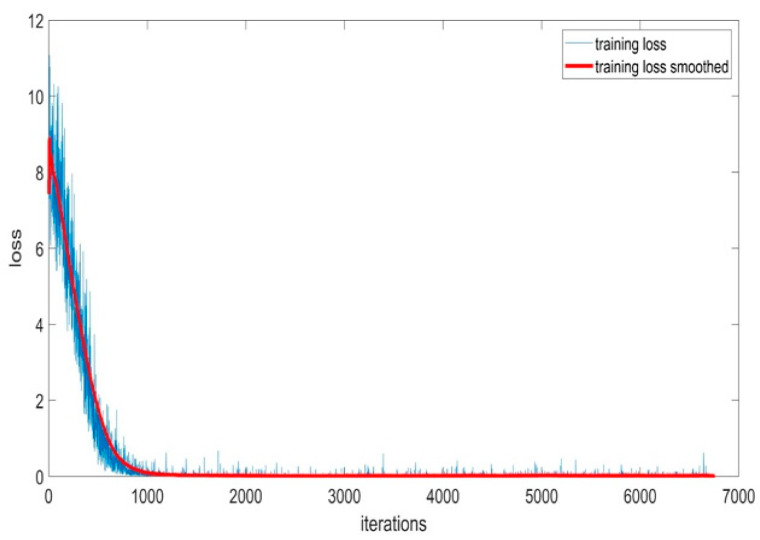
Training Loss Accuracy for BaysFusCNN Model.

Figure 20 illustrates the Training Loss Accuracy and Smoothed Training Loss Accuracy of the BaysFusCNN model during its 250 training epochs. The *x*-axis represents the epochs, and the *y*-axis shows the loss score, ranging from 0 to 8.

The blue line represents Training Loss Accuracy, which consistently decreases as the model learns, indicating improved prediction and reduced error. The orange line represents Smoothed Training Loss Accuracy, calculated using a moving average window, providing a smoother depiction of the model’s loss reduction.

**Figure 21:**
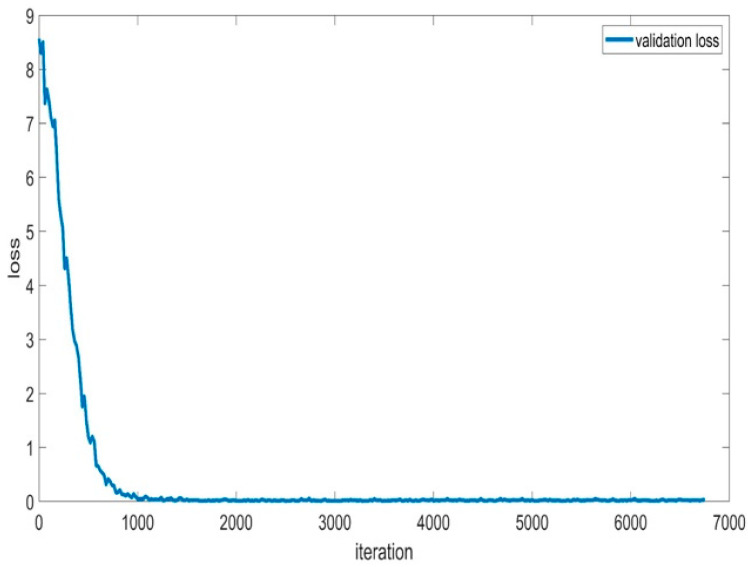
Validation Loss Accuracy for BaysFusCNN Model.

In Figure 21, the validation loss accuracy is consistently lower than the training loss accuracy, demonstrating that the model generalizes well to new data and avoids overfitting. The gradual decrease in validation loss accuracy over time showcases the model’s ability to minimize loss and enhance prediction accuracy.

Comparing validation loss accuracy (Figure 20) to training loss accuracy (Figure 21) reveals that the model consistently outperforms unseen data. The diminishing gap between the two curves signifies continuous improvement without overfitting. Overall, these figures confirm the BaysFusCNN model’s capacity to learn, minimize loss, and generalize effectively to new data.

### 5.3. Comparing BaysFusCNN & BaysCNN for DDoS Detection in Cloud Environment

The BaysFusCNN model demonstrates significant enhancements compared to the BaysCNN model, marking a substantial improvement in various critical aspects of DDoS attack detection in cloud environments. Despite similar training accuracy, the BaysFusCNN model excels in addressing class imbalances, particularly for minority classes such as DrDoS_LDAP and UDP-lag, where it outperforms the BaysCNN model. This fusion approach proves highly beneficial for countering dataset imbalances and improving overall detection capabilities. Key findings of the comparison include:

1. Accuracy: The BaysFusCNN model achieves an average accuracy of 99.79%, a notable improvement over the BaysCNN model’s 99.66%. This increase highlights the advantages of combining multiple classifiers for enhanced network attack detection accuracy.
2. True Positive Recall Sensitivity: In most classes, the BaysFusCNN model outperforms the BaysCNN model in True Positive Recall Sensitivity, particularly in classes like DrDoS_LDAP and DrDoS_NTP. This indicates the fusion model’s superior ability to identify previously misclassified instances, a crucial aspect of network security.
3. Predicted Positive Precision: The BaysFusCNN model demonstrates superior Predicted Positive Precision in most classes, emphasizing its proficiency in recognizing true positive cases. This reduces false alarms and improves the model’s precision in distinguishing actual threats.
4. Actual Negative Specificity: The BaysFusCNN model significantly enhances actual negative Specificity across most classes compared to the BaysCNN model. This reduction in false positives ensures accurate identification of benign traffic, a vital aspect of network security.
5. FP Recall: The BaysFusCNN model achieves a lower FP Recall rate (0.12%) compared to the BaysCNN model (0.18%), indicating a reduced rate of false positives and enhanced accuracy in identifying genuine threats.
6. F1 Score: The BaysFusCNN model achieves an F1 Score of 98.56%, outperforming the BaysCNN model’s F1 Score of 97.66%. This balanced metric highlights the fusion model’s improved overall performance.

In summary, the BaysFusCNN model’s enhancements, including accuracy, True Positive Recall Sensitivity, Predicted Positive Precision, actual negative Specificity, reduced FP Recall, and F1 Score, demonstrate the benefits of combining multiple classifiers for network attack detection and its potential to strengthen network security. For a detailed comparison of metrics between the BaysCNN and BaysFusCNN models, please refer to Table 10.

### 5.4. Comparing BaysFusCNN and Other Similar Models

Table 11 provides a comprehensive comparison of BaysFusCNN with other similar models, highlighting BaysFusCNN’s superior performance in various key metrics:

BaysFusCNN achieves the highest accuracy rate of 99.79%, surpassing the other two models, which achieved accuracy rates of 97.90% and 98.15%, respectively. Additionally, BaysFusCNN outperforms the other models in terms of true positive recall, predicted positive precision, and F1 Score metrics, indicating its superior ability to accurately identify true DDoS attacks from the positive samples. Specifically, BaysFusCNN achieves a true positive recall sensitivity of 98.55%, a predicted positive precision of 99.88%, and an F1 Score of 98.56%, all of which are higher than the corresponding values reported by the other models.

While BaysFusCNN has a slightly lower actual negative specificity value than the other models, its stronger performance in the other metrics more than compensates for this difference. Therefore, it can be concluded that BaysFusCNN consistently outperforms other similar models in the task of detecting DDoS attacks, making it a highly effective choice for DDoS detection in a cloud environment. 

## 6. Conclusions

In conclusion, this research addresses critical cybersecurity challenges in cloud environments by introducing two innovative models, BaysCNN and Data Fusion BaysFusCNN. BaysCNN demonstrates exceptional performance with an average accuracy of 99.66%, showcasing its effectiveness in detecting and mitigating various types of DDoS attacks.

The integration of Bayesian techniques and feature fusion in the Data Fusion BaysFusCNN approach yields even higher accuracy, reaching an impressive 99.79% for multi-class attacks. This demonstrates the model’s superior capabilities in identifying and responding to complex security threats. These findings provide valuable insights for organizations seeking to bolster their cloud and network security. By adopting these approaches, businesses can significantly enhance the reliability and scalability of their intrusion detection systems. Effective threat detection not only safeguards critical data but also empowers organizations to leverage the advantages of cloud computing confidently.

In summary, this research advances the field of machine learning-based intrusion detection systems, offering a promising path forward in securing cloud-based environments. As cloud technology adoption continues to grow, the proposed models contribute to the evolving landscape of cybersecurity, providing robust protection against emerging threats.

## 7. Future Work

To enhance the BaysFusCNN model’s robustness in detecting DDoS attacks in cloud environments, future work should focus on evaluating its performance in the presence of different types and levels of noise. This can be achieved through steps like noise generation, creating noisy datasets, and assessing the model’s performance in various noisy conditions. This research will provide insights into improving the model’s resilience in real-world scenarios where data often contains noise or distortions, ensuring its effectiveness in practical applications.

## Figures and Tables

**Table 1:** Comparative Analysis of Previous Studies.

Ref.	Year	Description	Dataset	Strengths	Limitations
[11]	2006	Utilized DT and RNN for intrusion detection on the KDD dataset.	KDD	94% accuracy in attack detection.	Struggled with detecting 15% of new attack patterns.
[12]	2019	Developed DNN and LSTM models for predicting DoS and DDoS attacks using CICIDS2017.	CICIDS2017	98.72% accuracy for DNN and 96.15% for LSTM.	Limited real-time ca- abilities.
[13]	2019	Introduced VCDeepFL technique for identifying DDoS attacks using an outdated dataset.	NSL-KDD	99.3% accuracy for normal traffic.	Encountered challenges in identifying new and unknown attacks.
[14]	2019	Developed LSTM-BA model, combining LSTM and Bayes techniques for detecting DDoS attacks.	ISCX2012	Accuracy = 98.15%, Precision = 98.42%, Recall = 97.6%.	Slower attack detection speed.
[15]	2015	Employed Bagging Ensemble framework with Partial Decision Trees for intrusion detection.	KDD Cup ‘99	99.71% classification accuracy.	Extended model construction times.
[16]	2020	Introduced a multi-objective optimization approach, combining CNN and LSTM for intrusion detection.	CICIDS2017	99.03% accuracy and 99.36% F1 Score.	Lacked information about attack characteristics.
[17]	2018	Proposed hybrid model with DBN and SVM for distributed network intrusion detection.	KDD Cup ‘99, NSL-KDD, UNSW-NB15, CICIDS2017	High performance in finding suspicious activities.	Increased complexity in model construction.
[18]	2024	Inverse chi square-based flamingo search optimization with machine learning-based security solution for IoT edge devices.	anomaly database	Achieves a commendable 98.25% accuracy in threat recognition for IoT edge devices. Provides an automated security solution, addressing IoT edge device security concerns.	Dataset information lacks details

**Table 2:** CICDDoS2019 Dataset Class Distribution.

Class	Samples
TFTP	20,082,580
DrDoS_SNMP	5,159,870
DrDoS_DNS	5,071,011
DrDoS_MSSQL	4,522,492
DrDoS_NetBIOS	4,093,279
DrDoS_UDP	3,134,645
DrDoS_SSDP	2,610,611
DrDoS_LDAP	2,179,930
Syn	1,582,289
DrDoS_NTP	1,202,642
UDP-lag	366,461
WebDDoS	439
Portmap	186,960
BENIGN	56,863

**Table 3:** CICDDoS2019 Dataset Class Distribution After Sampling.

Class	Samples
TFTP	44,000
DrDoS_SNMP	44,000
DrDoS_DNS	88,000
DrDoS_MSSQL	44,000
DrDoS_NetBIOS	44,000
DrDoS_UDP	44,000
DrDoS_SSDP	44,000
DrDoS_LDAP	44,000
Syn	44,000
DrDoS_NTP	44,000
UDP-lag	44,000
BENIGN	38,129

**Table 4:** Hardware Specification Used to Conduct Experiment.

Component	Specifications
Processor	2.3 GHz 8-core 9th-generation Intel Core i9 processor with Turbo Boost up to 4.8 GHz
RAM	16 GB of DDR4 memory
GPU	AMD Radeon Pro 5500M with 4 GB of GDDR6 memory
Storage	1 TB SSD storage
Display	16-inch Retina display with a resolution of 3072 by 1920 pixels
Ports	Four Thunderbolt 3 (USB-C) ports, a headphone jack, and an SDXC card slot

**Table 5:** Training Options Used to Conduct Experiment.

Option	Meaning
“Adam”	Optimizer used during training.
gradientdecayfactor = 0.9000	Decay factor used for the learning rate.
squaredgradientdecayfactor = 0.9900	Decay factor for the second moment estimate used in Adam optimizer.
Epsilon = 5 × 104	A small value is added to the denominator to avoid division by zero.
initiallearnrate = 0.001	The learning rate used by the optimizer at the start of training.
learnrateschedule = ‘none’	The learning rate is not adjusted during training.
learnratedropfactor = 0.1000	The learning rate is reduced by 10
L2Regularization = 1.0000 × 104	Strength of the L2 regularization applied to the model.
gradientthresholdmethod = ‘l2 norm’	The maximum l2norm of the gradient will be used as the threshold.
gradientthreshold = Inf	The gradient threshold is set to infinity, meaning there is no threshold.
maxepochs = 250	The maximum number of epochs the model can train for.
minibatchsize = 10,000	The number of samples used in each batch during training.
Verbose = 1	Some output information is displayed in the console during training.
verbosefrequency = 50	The output information is displayed after every 50 epochs.
validationdata = Vdata, vpredictors	The validation dataset is held in a cell array Vdata, vpredictors.
validationfrequency = 20	Validation metrics are evaluated every 20 epochs.
validationpatience = Inf	Training will only stop when the validation loss does not improve any further.
Shuffle = ‘everyepoch’	Input batches are shuffled every epoch.
checkpointpath = “ “	Training checkpoints are not saved.
executionenvironment = ‘auto’	The software selects the best available hardware device.
sequencelength = ‘longest’	The input sequences are padded to their longest length.
sequencepaddingvalue = 0	The padding value used for the input sequences is zero.
sequencepaddingdirection = ‘right’	The padding is executed on the right end of the sequences.
dispatchinbackground = 0	The training job is not dispatched in the background.
resetinputnormalization = 1	Input normalization is reset before starting training.
batchnormalizationstatistics = ‘population’	The full dataset is used when computing batch normalization statistics.
outputnetwork = ‘lastiteration’	The trained network is output after the final epoch of training.

**Table 6:** The Results of BaysCNN.

Class	Accuracy	TP Recall	PP Precision	AN Specificity	FP Recall	F1 Score
Benign	99.66%	97.26%	97.35%	99.82%	0.18%	97.30%
DrDoS_DNS	99.84%	99.67%	99.20%	99.86%	0.14%	99.43%
DrDoS_LDAP	99.54%	95.01%	98.55%	99.89%	0.11%	96.74%
DrDoS_MSSQL	99.73%	98.59%	97.62%	99.81%	0.19%	98.10%
DrDoS_NTP	99.51%	95.67%	97.45%	99.80%	0.19%	96.55%
DrDoS_NetBIOS	99.82%	100%	97.54%	99.80%	0.19%	98.76%
DrDoS_SNMP	99.67%	97.90%	97.53%	99.80%	0.19%	97.71%
DrDoS_SSDP	99.75%	98.58%	98.03%	99.84%	0.15%	98.31%
DrDoS_UDP	99.70%	98.82%	97.17%	99.78%	0.22%	97.99%
Portmap	99.43%	95.06%	97.09%	99.78%	0.22%	96.06%
Syn	99.46%	95.17%	97.41%	99.80%	0.20%	96.28%
TFTP	99.78%	99.16%	97.76%	99.83%	0.17%	98.46%
UDP-lag	99.70%	98.64%	97.23%	99.78%	0.22%	97.93%
averages	99.66%	97.66%	97.69%	99.82%	0.18%	97.66%

**Table 7:** Confusion Matrix Results for BaysCNN.

Class	True Positives TP	False Negatives FN	False Positives FP
Class 1	11,223	316	306
Class 2	26,200	88	212
Class 3	12,601	662	186
Class 4	12,986	186	317
Class 5	12,604	571	330
Class 6	13,108	0	330
Class 7	12,899	277	327
Class 8	13,065	188	262
Class 9	13,070	156	380
Class 10	12,691	659	381
Class 11	12,619	641	335
Class 12	12,931	109	296
Class 13	13,009	180	371

**Table 8:** The Results of BaysFusCNN.

Class	Accuracy	TP Recall	PP Precision	AN Specificity	FP Recall	F1 Score
Benign	99.87%	99.49%	98.42%	99.89%	0.11%	98.95%
DrDos_DNS	99.82%	99.61%	99.17%	99.86%	0.14%	99.39%
DrDos_LDAP	99.78%	97.95%	99.01%	99.92%	0.08%	98.48%
DrDos_MSSQL	99.84%	98.80%	98.94%	99.92%	0.08%	98.87%
DrDos_NTP	99.65%	97.54%	97.53%	99.81%	0.19%	97.53%
DrDos_NetBIOS	99.80%	98.70%	98.61%	99.89%	0.11%	98.66%
DrDos_SNMP	99.83%	99.29%	98.33%	99.87%	0.13%	98.81%
DrDos_SSDP	99.77%	98.60%	98.35%	99.87%	0.13%	98.48%
DrDos_UDP	99.76%	97.89%	98.76%	99.90%	0.10%	98.32%
Portmap	99.69%	97.30%	98.43%	99.88%	0.12%	97.86%
Syn	99.79%	98.76%	98.38%	99.87%	0.13%	98.57%
TFTP	99.83%	98.82%	98.89%	99.91%	0.09%	98.85%
UDP-lag	99.79%	98.41%	98.62%	99.89%	0.11%	98.51%
Averages	99.79%	98.55%	98.57%	99.88%	0.12%	98.56%

**Table 9:** The Results of Confusion Matrix for BaysFusCNN.

Class	True Positives TP	False Negatives FN	False Positives FP
Class 1	11,308	58	181
Class 2	26,175	103	219
Class 3	12,879	269	129
Class 4	12,955	157	139
Class 5	12,823	324	325
Class 6	13,279	174	187
Class 7	13,048	93	221
Class 8	13,073	185	219
Class 9	13,063	282	164
Class 10	12,795	355	204
Class 11	12,974	163	213
Class 12	13,208	158	148
Class 13	12,929	209	181

**Table 10:** Comparison of Metrics between BaysCNN and BaysFusCNN Models.

Metric	BaysCNN Model (%)	BaysFusCNN Model (%)
Accuracy	99.66%	99.79%
TP Recall	97.66%	98.55%
PP Precision	97.69%	98.57%
AN Specificity	99.82%	99.88%
FP Recall	0.18%	0.12%
F1 Score	97.66%	98.56%

**Table 11:** Comparing BaysFusCNN and other Similar Models.

Model	Accuracy	TP Recall	PP Precision	AN Specificity	FP Recall	F1 Score
Spiking Neural Network (SNN) (2020) [16]	99.03%	-	-	-	-	99.36%
Combine LSTM + Bayes techniques (2019) [14]	98.15%	97.6%	98.42%	98.4%,	1.6%	98.05%
ICSFSO-ML (2024) [18]	98.22%	98.22%	98.25%	-	-	98.22%
BaysFusCNN	99.79%	98.55%	98.57%	99.88%	1.2%	98.56%

## Data Availability

The dataset supporting the findings of this study are derived from previously published datasets, which have been cited within the manuscript. Available online: https://www.unb.ca/cic/datasets/ddos-2019.html (accessed on 23 December 2023).
